# A proposal of a perfect graphene absorber with enhanced design and fabrication tolerance

**DOI:** 10.1038/s41598-017-04995-w

**Published:** 2017-07-06

**Authors:** Sangjun Lee, Thang Q. Tran, Hyungjun Heo, Myunghwan Kim, Sangin Kim

**Affiliations:** Department of Electrical and Computer Engineering, Ajou University, Suwon, South Korea

## Abstract

We propose a novel device structure for the perfect absorption of a one-sided lightwavve illumination, which consists of a high-contrast grating (HCG) and an evanescently coupled slab with an absorbing medium (graphene). The operation principle and design process of the proposed structure are analyzed using the coupled mode theory (CMT), which is confirmed by the rigorous coupled wave analysis (RCWA). According to the CMT analysis, in the design of the proposed perfect absorber, the HCG, functioning as a broadband reflector, and the lossy slab structure can be optimized separately. In addition, we have more design parameters than conditions to satisfy; that is, we have more than enough degrees of freedom in the device design. This significantly relieves the complexity of the perfect absorber design. Moreover, in the proposed perfect absorber, most of the incident wave is confined in the slab region with strong field enhancement, so that the absorption performance is very tolerant to the variation of the design parameters near the optimal values for the perfect absorption. It has been demonstrated numerically that absorption spectrum tuning over a wider wavelength range of ~300 nm is possible, keeping significantly high maximum absorption (>95%). It is also shown that the proposed perfect absorber outperforms the previously proposed scheme in all aspects.

## Introduction

**Figure 1:**
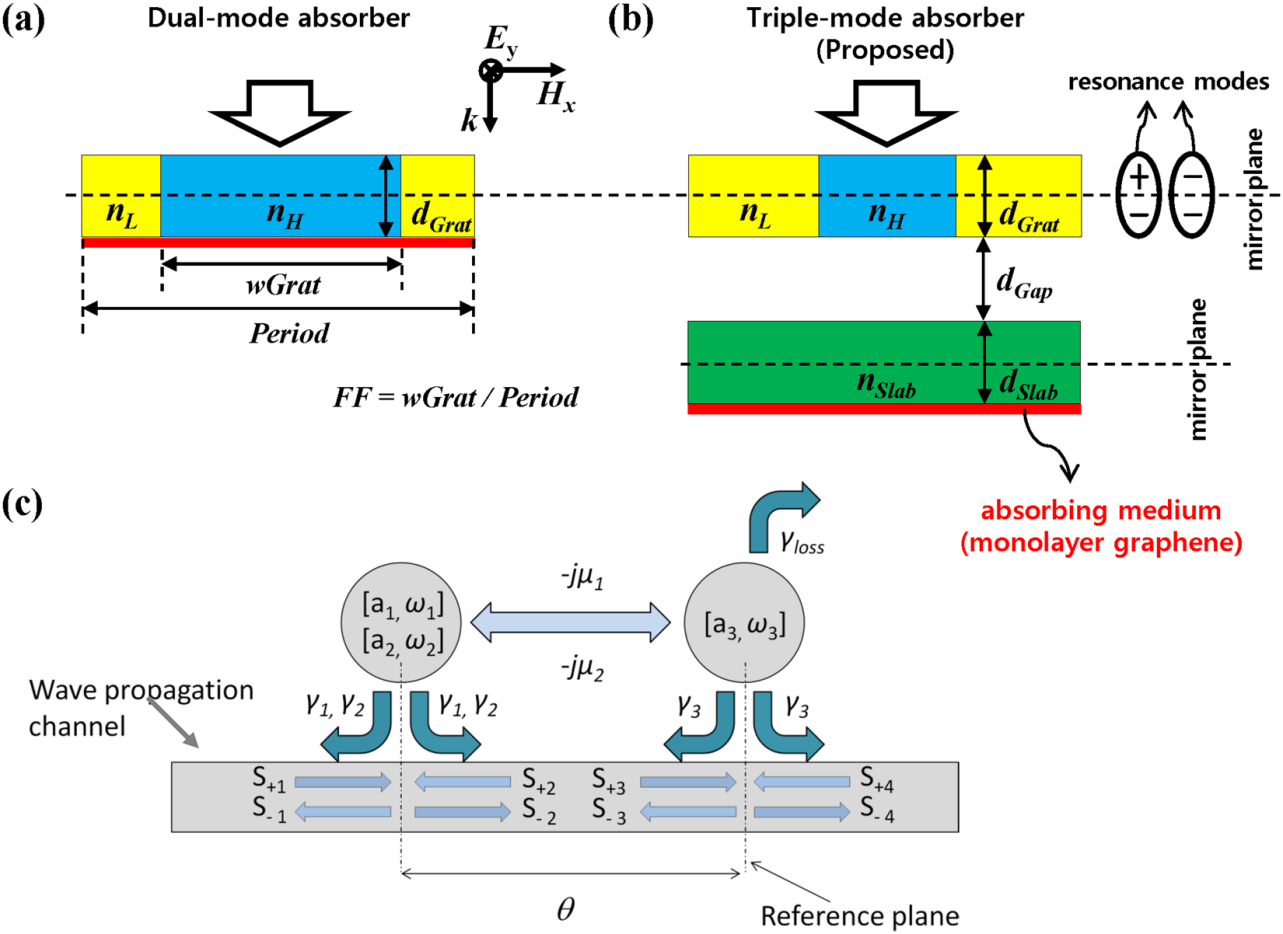
(**a**) Schematic of the previously proposed “*dual*-*mode absorber*” using an HCG, assuming that *n*_*H*_ = 3.0, *n*_*L*_ = 1.5, *Period* = 1.0 μm, and *FF* = 0.946 unless otherwise stated. *FF* is defined as the ratio of higher-index material width (*wGrat*) to *Period*. (**b**) Schematic of the proposed “*triple*-*mode absorber*”, assuming that *n*_*H*_ = 3.0, *n*_*L*_ = 1.5, *Period* = 0.9 μm, and *FF* = 0.43 unless otherwise stated. In both absorbers, the HCG supports two resonance modes with opposite symmetry with respect to the mirror plane perpendicular to the incident light. (**c**) Theoretical model of the proposed “*triple*-*mode absorber*” for the coupled mode theory analysis, where the slab with a graphene layer is treated as a high-Q resonator (a_3_) with loss, and the HCG is assumed to support two nondegenerate resonant modes (a_1_ and a_2_) without loss, and both direct and indirect couplings between the HCG and the slab are considered. The red thin layers in (**a**) and (**b**) indicate monolayer graphene of *t*_*G*_ = 0.34 nm thickness as an absorbing medium.

Undoped monolayer graphene has high conductivity and exhibits broadband light absorption of 2.3% over a range from terahertz to the visible light frequency. Owing to its unique electronic and optical properties, graphene has attracted strong interest in developing high-speed graphene-based photodetectors^1–6^. Considering its atomically ultrathin thickness of ~0.34 nm, the absorption of graphene is rather high. However, for practical high-performance photodetectors, the absorption should be enhanced considerably. To enhance the absorption in the ultrathin absorbing layer, a resonant structure such as a grating or a photonic crystal can be used. However, the maximum achievable absorption in a single-mode resonator-based structure is 50%, so that a sophisticated design of the resonant structure is required to obtain 100% absorption. Recently, several schemes for 100% absorption in graphene under one-side illumination have been proposed, which can be classified into two categories. One uses single resonance mode and a perfect mirror^7–11^. Usually, an ideal metallic reflector or a dielectric Bragg reflector is used as the back mirror, and gratings or photonic crystal slabs play a role as a resonator. In this “*single*-*mode/mirror absorber*”, a perfect absorption condition is rather simple: the leakage rate of the resonator should be equal to its loss rate, which is a so-called “*critical coupling*” condition. However, the metallic reflector causes unwanted loss, and the Bragg reflector requires a rather complicated fabrication of several tens of films. The other category uses two degenerate resonance modes with opposite symmetry, avoiding the use of a mirror, where each resonance is *critically coupled* with the incident wave and is responsible for 50% absorption, resulting in a total of 100% absorption: this is called a “*dual*-*mode* (or *Degenerate critical coupling*) *absorber*”^12^. In the “*dual*-*mode absorber*” (Fig. 1(a)), perfect absorption in graphene can be achieved without a perfect mirror, but in reality, it is very difficult to achieve because the frequency degeneracy and the critical coupling conditions of dual modes must be simultaneously satisfied, which could be prohibitively restrictive. Indeed, in ref. 12 a peak absorption of 98% was reported owing to residual 2% scattering due to imperfect satisfaction of the degeneracy and the critical coupling conditions. Thus, this scheme inevitably has poor tolerance for geometric parameters and graphene quality.

In this work, we propose a novel scheme to achieve perfect graphene absorption more simply and provide much wider absorption tunability compared to the previously proposed “*dual*-*mode absorber*” (Fig. 1(a)). The proposed absorber (Fig. 1(b)) consists of a high-contrast grating (HCG) with a broadband reflection spectrum^13–16^, a slab separated by a gap region, and monolayer graphene placed just below the slab. All background regions including the gap are filled with vacuum. Although the proposed structure includes HCG similarly to the “*dual*-*mode absorber*”, its perfect absorption mechanism is completely different from the “*dual*-*mode absorber*”. In our proposed structure, perfect absorption is achieved through the coupling among three modes—two (dual) low-Q HCG modes and a single high-Q slab mode—and the frequency degeneracy condition is no longer required. Hereafter, our proposed scheme is dubbed “*triple*-*mode absorber*”. Our theoretical investigation reveals that the enhanced design and fabrication tolerance of the proposed structure is attributed to the interaction among three resonant modes. To analyze the conditions for the perfect absorption in the “*triple*-*mode absorber*”, we first perform coupled mode theory (CMT) analysis^17–19^ and then present several numerical calculations using the rigorous coupled wave analysis (RCWA) method^20^. The excellent agreement between the CMT calculation and the numerical simulation validates our theoretical model of the “*triple*-*mode absorber*”.

## Results

### Design of the “triple-mode absorber”

Figure 1(c) shows the theoretical model of the proposed “*triple*-*mode absorber*” for the CMT analysis. The HCG is treated as a lossless resonator with two nondegenerate resonance modes with leakage rates *γ*_1_ and *γ*_2_ and resonance frequencies *ω*_1_ and *ω*_2_, respectively. For each resonance mode of the HCG, the leakage rate is defined as the ratio of resonance frequency to 4Q, where Q is the quality factor, and it is related to an index modulation of the HCG. The slab with a graphene layer is treated as a lossy single-mode resonator with a leakage rate *γ*_3_, a loss rate *γ*_*l*oss_, and a resonance frequency *ω*_3_. In general, a slab waveguide alone cannot be considered as a resonator. In the proposed structure (Fig. 1(b)), however, the top HGC works as a scattering source, so that wavelength-selective wave coupling to the lower slab waveguide—so-called guided-mode resonance—is allowed, and the slab can be treated as a resonator with a low leakage rate—that is, high-Q. *μ*_1_ and *μ*_2_ are the direct (or evanescent) coupling strengths between the modes of the first resonator and that of the second resonator, respectively. *s*_+*i*_ and *s*_−*i*_ are the amplitudes of the incoming and outgoing waves, respectively, with respect to the *i* ^th^ resonator. *θ* is the propagation-induced phase difference between the two resonators. We assume that both resonators have mirror symmetry and the two resonance modes of the first resonator have opposite symmetry, as schematically shown in Fig. 1(a–b). From the time-domain coupled mode equations, transmission and reflection coefficients can be calculated in a frequency domain^17–19^. By setting both transmission and reflection coefficients of the system to zero, at the resonance frequency *ω* = *ω*_*0*_, we obtained the following perfect absorption conditions (Supplementary Information):

1 $$({\omega }_{0}-{\omega }_{1})({\omega }_{0}-{\omega }_{2})+{\gamma }_{1}{\gamma }_{2}=0,$$

2 $$\theta =(2n+1)\pi ,$$

3 $$\sqrt{{\gamma }_{3}}=\frac{{\mu }_{1}({\omega }_{0}-{\omega }_{2})+{\mu }_{2}\sqrt{{\gamma }_{1}{\gamma }_{2}}}{2{\gamma }_{2}\sqrt{{\gamma }_{1}}},$$

4 $${\gamma }_{loss}=\frac{-2{\mu }_{1}{\mu }_{2}\sqrt{{\gamma }_{1}{\gamma }_{2}}({\omega }_{0}-{\omega }_{2})+2{\gamma }_{1}{{\gamma }_{2}}^{2}{\gamma }_{3}}{{\gamma }_{1}({({\omega }_{0}-{\omega }_{2})}^{2}+{{\gamma }_{2}}^{2})},$$

5 $${\omega }_{3}={\omega }_{0}-\frac{({\omega }_{0}-{\omega }_{2})({{\mu }_{2}}^{2}{\gamma }_{1}-{{\mu }_{1}}^{2}{\gamma }_{2}-2{\gamma }_{1}{\gamma }_{2}{\gamma }_{3})}{{\gamma }_{1}({({\omega }_{0}-{\omega }_{2})}^{2}+{{\gamma }_{2}}^{2})}.$$

It is interesting to note that equation (1) originates from zero transmission constraint, and the resonance (perfect absorption) frequency of the system *ω*_*0*_ is solely determined by the design parameters of the first resonator (*ω*_1_, *ω*_2_, *γ*_1_, and *γ*_2_), all of which are independent of the design for the second resonator. Equation (1) is actually equivalent to the condition for zero transmission (100% reflection) in the lossless resonator system supporting two resonance modes of opposite symmetries in refs 12, 17 and 18 For given parameters of the first resonator and a given loss rate (*γ*_loss_), we have three equations (3–5) to satisfy, apart from (2), with 4 variables (*ω*_3_, *μ*_1_, *μ*_2_, and *γ*_3_). Thus, we can always realize perfect absorption with the “*triple*-*mode absorber*” structure. Moreover, from equations (3–5), one can consider the optimal leakage rate (γ_3_) and resonance frequency of the second resonator (*ω*_3_) for the perfect absorption as functions of direct coupling strengths (*μ*_1_ and *μ*_2_), assuming that the remaining parameters are predetermined. This viewpoint is quite useful in designing a real perfect or high-performance absorber as discussed later.

If we assume that two solutions of (1) are close enough to form a broadband reflector^18^, we obtain

6 $$({\omega }_{0}-{\omega }_{2})\approx -\sqrt{{\gamma }_{1}{\gamma }_{2}}.$$

Because the leakage rate of the slab waveguide must be very small in the proposed absorber structure as mentioned earlier, we can reasonably assume

7 $${\gamma }_{3}\approx 0.$$

Actually, *γ*_3_ is several orders of magnitude less than *γ*_1_ and *γ*_2_, as shown later. Substituting (6) and (7) into (3), we obtain

8 $${\mu }_{1}\approx {\mu }_{2}.$$

This appears reasonable because the distances between the two modes of the first resonator and the second resonator mode are identical. Substituting (6) and (7) into (4), we obtain

9 $${\gamma }_{loss}\approx 2{\mu }_{1}{\mu }_{2}/({\gamma }_{1}+{\gamma }_{2}).$$

By substituting (6), (7), and (8) into (5), we obtain

10 $${\omega }_{3}-{\omega }_{0}\approx \frac{{\mu }_{1}^{2}({\gamma }_{1}-{\gamma }_{2})}{\sqrt{{\gamma }_{1}{\gamma }_{2}}({\gamma }_{1}+{\gamma }_{2})}.$$

One can see that (9) can be satisfied by properly choosing the direct coupling strengths (*μ*_1_ and *μ*_2_) for any given loss rate (*γ*_loss_) and the first resonator parameters. Once the proper coupling strengths are chosen, the proper resonance frequency of the slab mode is determined by (10). This implies that the design of the perfect or high-performance “*triple*-*mode absorber*” simply turns into the design of the resonance frequency of the slab and the direct coupling strengths.

Because we are interested in the case when the loss late of the absorbing medium is much less than the leakage rates of the first resonator (*γ*_loss_ ≪ *γ*_1_ and *γ*_2_), where a special design of absorption-enhancing structure is required, (9) implies *μ*_1_, *μ*_2_ ≪ *γ*_1_, *γ*_2_; we then obtain ${\omega }_{3}-{\omega }_{0}\approx 0$ from (10).

**Figure 2:**
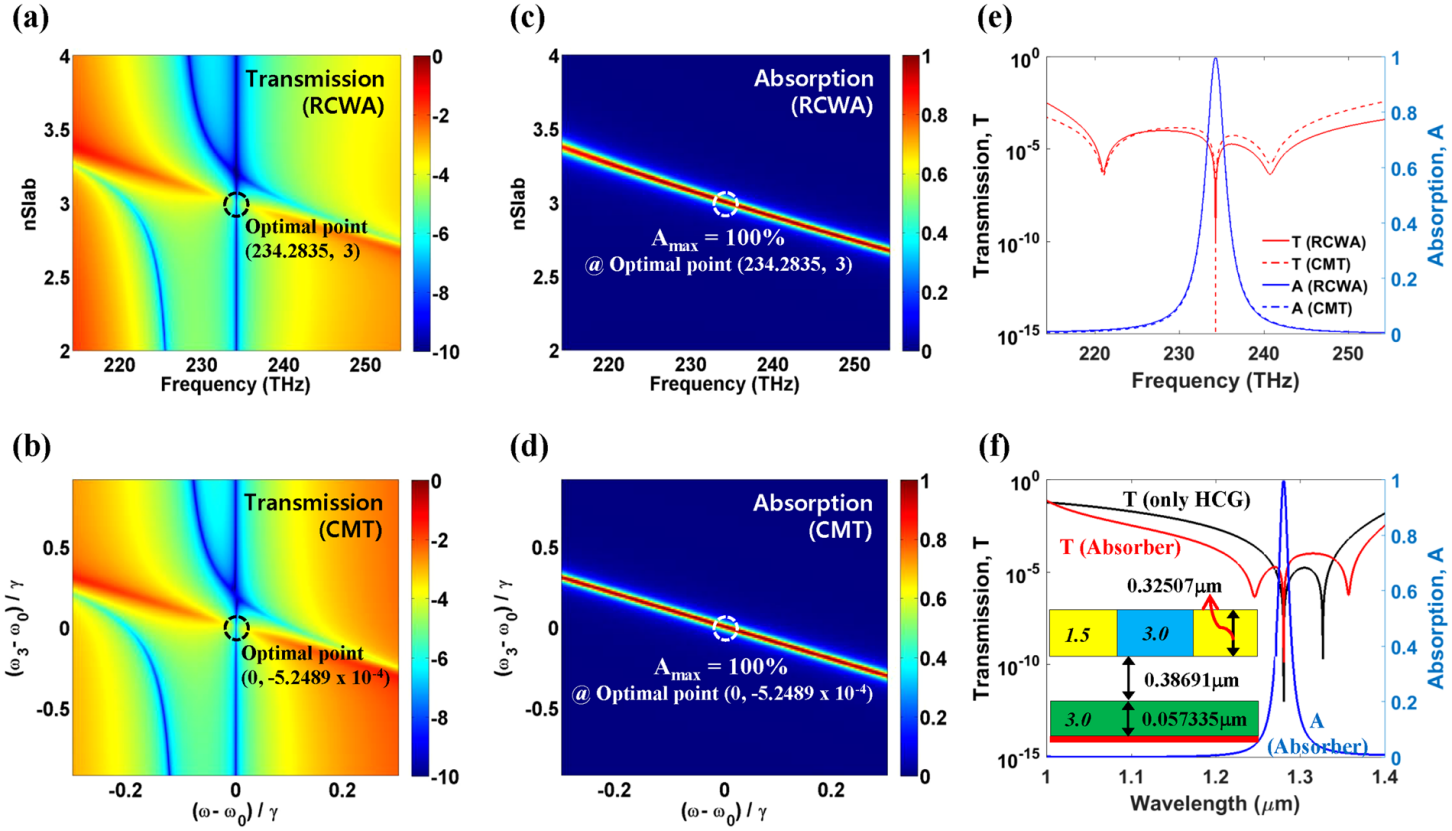
Transmission spectra calculated using (**a**) RCWA and (**b**) CMT for the proposed “*triple*-*mode absorber*” with *d*_*Grat*_ = 0.32507 μm, *d*_*Gap*_ = 0.38691 μm, and *d*_*Slab*_ = 0.057335 μm. Both are plotted on the same logarithmic color scale. Absorption spectra calculated using (**c**) RCWA and (**d**) CMT for the same conditions. In all plots, the optimal (perfect absorption) points are also denoted by the dashed circle. (**e**) Comparison of RCWA and CMT results for each optimal condition; that is, *n*_*Slab*_ = 3 and (*ω*_3_ *− ω*_0_)/*γ* = −5.2489 × 10^−4^. (**f**) Comparison of RCWA results for the optimal “*triple*-*mode absorber*” (*n*_*Slab*_ = 3) and a lossless HCG alone (the inset indicates the schematic of the designed perfect “*triple*-*mode absorber*”).

**Figure 3:**
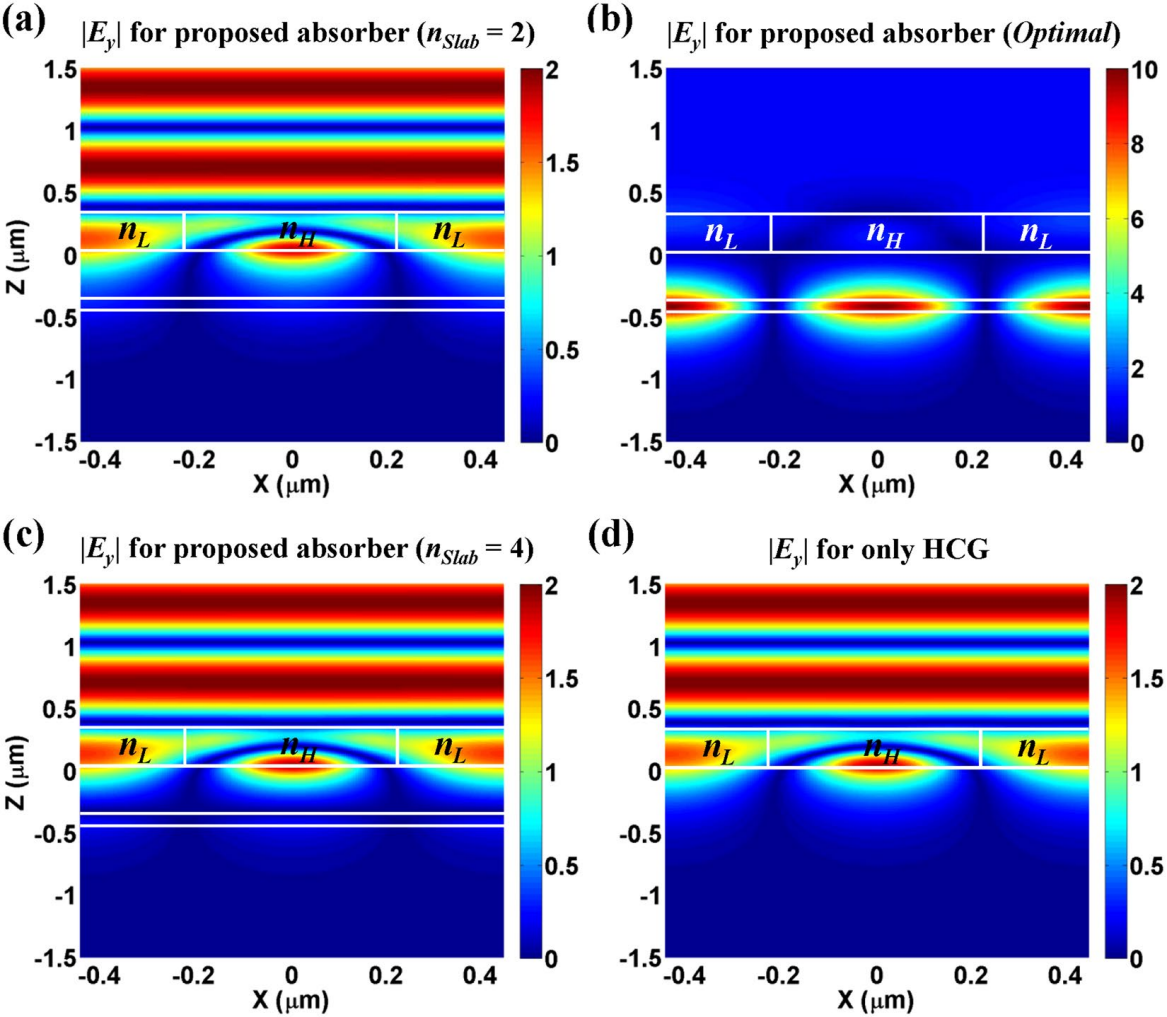
Electric field distributions (|*E*_*y*_|) at *λ* = 1.2805 μm for the proposed “*triple*-*mode absorber*” with (**a**) *n*_*Slab*_ = 2, (**b**) *n*_*Slab*_ = 3 (optimal), (**c**) *n*_*Slab*_ = 4, and for (**d**) a lossless HCG alone. Note that the remaining parameters are the same as those of the inset in Fig. 2(f); that is, *d*_*Grat*_ = 0.32507 μm, *d*_*Gap*_ = 0.38691 μm, and *d*_*Slab*_ = 0.057335 μm, and all calculations are conducted using RCWA.

The validity of the CMT analysis and the design process is confirmed by simulation of a real device using RCWA. For the proposed structure under transverse electric (TE) wave illumination, the transmission spectra were calculated as a function of slab index (*n*_*Slab*_) using the RCWA and plotted in Fig. 2(a). We assumed that *d*_*Grat*_ = 0.32507 μm, *d*_*Gap*_ = 0.38691 μm, and *d*_*Slab*_ = 0.057335 μm. (All structural parameters are defined in Fig. 1(b). Note that remaining parameters are fixed unless otherwise stated; that is, *n*_*H*_ = 3.0, *n*_*L*_ = 1.5, *Period* = 0.9 μm, *FF* = 0.43, and the graphene thickness is 0.34 nm.) The complex permittivity of graphene (*ε*_*g*_) was extracted from the Kubo formula for the Fermi-level of *E*_*f*_ = 0 eV (undoped) and a mobility of *Mo* = 0.5 m^2^/Vs^21, 22^, unless otherwise stated. The spectra showed two transmission dips within the frequency range of our interest when *n*_*Slab*_ < ~2.7 or *n*_*Slab*_ > ~3.4. One of the dips exists at a frequency *f* = 234.2835 THz (*λ* = 1.2805 μm) irrespective of *n*_*Slab*_, whereas the other varies as *n*_*Slab*_ changes. Perfect absorption (A > 99.999999%) occurs at *f* = 234.2835 THz for *n*_*Slab*_ = 3, as marked by a dashed circle. Figure 2(b) shows the fitted transmission spectra calculated using the CMT as a function of (*ω*_3_ *− ω*_0_)/*γ*, where the fitting parameters are *ω*_1_ − *ω*_0_ = −0.9793*γ*, *ω*_2_ − *ω*_0_ = 0.8752*γ*, *γ*_1_ = *γ*, *γ*_2_ = 0.8571*γ*, *γ*_3_ = 2.0883 × 10^−6^ *γ*, *μ*_1_ = 0.1020*γ*, and *μ*_2_ = 0.0991*γ* (*γ* is an arbitrarily value of a unit of 1/*s*, and *ω*_*0*_ is the resonance frequency for the perfect absorption). The resonance frequency of the slab mode (*ω*_3_) is directly related to *n*_*Slab*_. On the whole, the trend of transmission spectra obtained from the CMT is almost identical to the RCWA result. When *ω*_3_ is far from the transmission dips of the first resonator, the interaction between two resonators is negligible, and the transmission dips of the whole system are determined solely by the first resonator. On the other hand, as *ω*_3_ becomes close to the transmission dips of the first resonator, there are three transmission dips within the frequency range of our interest, and one of the transmission dips of the first resonator is shifted. However, the other (main) transmission dip of the first resonator remains unchanged as *ω*_3_ varies. The perfect absorption point is also denoted by the dashed circle. One can see that the resonance frequency of the perfect absorption (*ω*_0_) is clearly independent of *ω*_3_ as expected from (1), and the perfect absorption occurs at the main transmission dips of the first resonator. The absorption spectra calculated using both methods are also plotted in Fig. 2(c) and (d), which also agree well with each other. Although perfect absorption occurs at the main transmission dip of the first resonator, the absorption peak is mainly determined by *ω*_3_ because most of the incident lightwave is confined in the second resonator as shown later (Fig. 3(b)).

The transmission and absorption spectra on the optimal (perfect absorption) conditions (*n*_*Slab*_ = 3 for the RCWA calculation, and *ω*_3_ − *ω*_0_ = −5.2489 × 10^−4^ *γ* for the CMT calculation), are plotted in Fig. 2(e). The frequencies of the transmission dips calculated using the CMT and the RCWA match exactly despite some discrepancy of the transmission value. The absorption spectrum calculated using the CMT agrees excellently with the numerical RCWA calculation result. The good agreement between the CMT and RCWA calculation results indicates that our theoretical model (in Fig. 1(c)) appropriately describes the behavior of our proposed “*triple*-*mode absorber*”.

To confirm that the perfect absorption frequency of the “*triple*-*mode absorber*” composed of the HCG and the lossy slab is determined solely by the main transmission dip frequency of the HCG, the transmission spectrum of the “*triple*-*mode absorber*” (red curve) is compared to that of the lossless HCG alone (black curve) in Fig. 2(f). The HCG structure was optimized to have an excellent flat-top high reflection (>99.99%) spectrum over the broadband wavelength ranges (*λ* = 1.26–1.34 μm). It is well known that the broadband reflection characteristic is based on destructive interference of dual modes with opposite symmetry^15, 18^. One can see that the main transmission dip frequency of the HCG is identical to the perfect absorption frequency of the “*triple*-*mode absorber*”. Regarding the other transmission dip (*λ* = 1.3269 μm) of the HCG, it can also be used for the design of the perfect absorber with different design parameters (*dGap* = 0.406 μm, *dSlab* = 0.06433 μm, and *nSlab* = 3.0), resulting in a maximum achievable absorption of *A* = 99.78%. We surmise that the lower absorption is attributed to the slightly higher transmission, corresponding to the lower reflection, compared to the main transmission dip. Thus, in the rest of this work, we focus on the design at *λ* = 1.2805 μm.

Figure 3(a),(b) and (c) show the field profiles calculated using RCWA in the “*triple*-*mode absorber*” at the perfect absorption frequency (*f* = 234.2835 THz, *λ* = 1.2805 μm) for *n*_*Slab*_ = 2, 3 (optimal), and 4, respectively. For comparison, the field profile in the HCG alone at the same frequency (wavelength) is shown in Fig. 3(d). When the resonance frequency of the slab is off-tuned (Fig. 3(a) and (c)), the field profiles of the “*triple*-*mode absorber*” are just the same as that in the case with HCG alone (Fig. 3(d)). This again verifies that the unchanged transmission dip of the “*triple*-*mode absorber*” is attributed to the main transmission dip of the HCG. In these cases, most of the incident wave is reflected, and the electric field enhancement in the HCG is very weak because of its low Q. On the other hand, when perfect absorption occurs, most of the field is confined in the slab with strong field enhancement (Fig. 3(c)), which results in perfect absorption by the atomically thin graphene layer placed just below the slab. One may wonder how the incident wave can reach the slab despite the zero transmission (100% reflection) of the HCG at the perfect absorption frequency. The dual modes of the HCG excited by the incident wave are partially coupled into the slab mode through the evanescent coupling, which are modeled as the direct coupling in the CMT (Fig. 1(c)). In addition, at the perfect absorption frequency, the scattered (reflected or transmitted) waves from the HCG are exactly canceled by the scattered wave from the slab. Thus, the entire incident wave is perfectly absorbed by the graphene layer.

**Figure 4:**
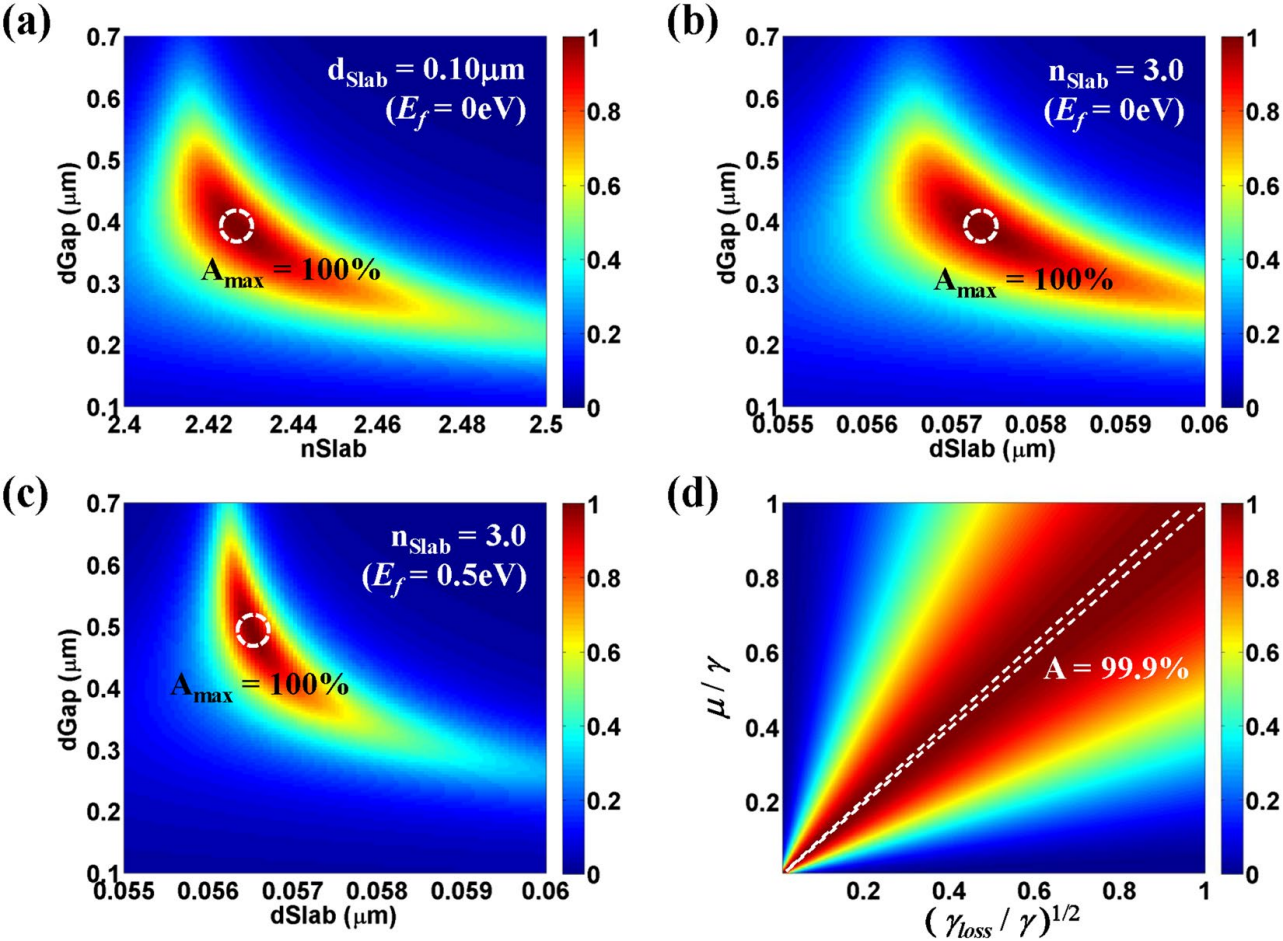
Various optimizations of absorption by RCWA at *λ* = 1.2805 μm for the proposed absorber (**a**) when *d*_*Slab*_ = 0.10 μm, *E*_*f*_ = 0 eV, (**b**) when *n*_*Slab*_ = 3.0, *E*_*f*_ = 0 eV, and (**c**) when *n*_*Slab*_ = 3.0, *E*_*f*_ = 0.5 eV, assuming that the remaining HCG parameters are the same as the optimal conditions considered in Fig. 2(f); that is, *n*_*H*_ = 3.0, *n*_*L*_ = 1.5, *Period* = 0.9 μm, *FF* = 0.43, and *d*_*Grat*_ = 0.32507 μm. (**d**) Absorption spectra by CMT as a function of loss rate and direct coupling strength, assuming that *μ* = *μ*_1_ = *μ*_2_ and the remaining fitting parameters are the same as the optimal condition in Fig. 2(b). Two dashed lines indicate A = 99.9%.

Once the high-performance HCG of flat-top high reflection (approximately 100%) over the broadband wavelength ranges is designed to have a main transmission dip at the desired frequency of perfect absorption, the next step to design the “*triple*-*mode absorber*” is to properly choose the slab parameters (*n*_*Slab*_ and *d*_*Slab*_) and the gap distance (*d*_*Gap*_) such that its resonance frequency fits the perfect absorption frequency and evanescent (direct) coupling strengths are optimal to a given loss rate of the absorbing material. As mentioned earlier, we have more variables than conditions to satisfy in this design step, so that the optimal choice of the design parameters (*n*_*Slab*_, *d*_*Slab*_, and *d*_*Gap*_) may not be unique. This will relieve the design complexity. To demonstrate this, for the same HCG as in the previous design (*n*_*H*_ = 3.0, *n*_*L*_ = 1.5, *Period* = 0.9 μm, *FF* = 0.43, and *d*_*Grat*_ = 0.32507 μm), two different approaches of the slab design were conducted at *λ* = 1.2805 μm: one with a fixed slab thickness of *d*_*Slab*_ = 0.1 μm (Fig. 4(a)) and the other with a fixed slab index of *n*_*Slab*_ = 3.0 (Fig. 4(b)). In both approaches, near-perfect absorption efficiencies (A > 99.999999%) were achieved. For the fixed slab thickness case, the optimal choice of the other parameters was *n*_*Slab*_ = 2.42666 and *d*_*Gap*_ = 0.38633 μm. For the fixed slab index, the optimal choice of the other parameters was *d*_*Slab*_ = 0.057335 μm, *d*_*Gap*_ = 0.38691 μm. Moreover, the design of the “*triple*-*mode absorber*” can be easily adapted to a loss rate change as discussed earlier on (4). Note that doping of graphene induces a decrease in the imaginary part of the permittivity of graphene (Supplementary Information) and thus a decrease in the loss rate of the lossy slab mode. For *E*_*f*_ = 0.5 eV, for instance, the loss rate decreases to ~1/3 of the undoped graphene’s value at *λ* = 1.2805 μm and *Mo* = 0.5 m^2^/Vs (Supplementary Information). To demonstrate the effect of doping, optimization of the slab was performed assuming *E*_*f*_ = 0.5 eV under the same conditions as in Fig. 4(b). The result is shown in Fig. 4(c). Near-perfect absorption (A > 99.999999%) was achieved for *d*_*Slab*_ = 0.05649115 μm and *d*_*Gap*_ = 0.48932 μm. This adaptive behavior of the “*triple*-*mode absorber*” to the loss rate variation is also expected from the CMT analysis. Figure 4(d) shows a plot of absorption as a function of the loss rate (*γ*_*loss*_) and the coupling strength (*μ*), where we assume that *μ* = *μ*_1_ = *μ*_2_ for the convenience of two-dimensional plotting, and the remaining fitting parameters are the same as the optimal condition in Fig. 2(b). Note that nearly perfect absorption can be achieved simply by tuning the direct coupling strength (*μ*) irrespective of *γ*_*loss*_. In designing the “*triple*-*mode absorber*”, the direct coupling strengths seem to be a dominant fitting factor from the CMT analysis, which is directly related to the real design parameters of *n*_*Slab*_, *d*_*Slab*_, and *d*_*Gap*_. In particular, *d*_*Gap*_ is the dominant parameter controlling the direct coupling strength *μ* because the overlap of field profiles of HCG modes and slab mode is inversely proportional to the distance between them; that is, *μ* is inversely proportional to *d*_*Gap*_.

### Excellent tolerance to structural parameters and graphene quality

**Figure 5:**
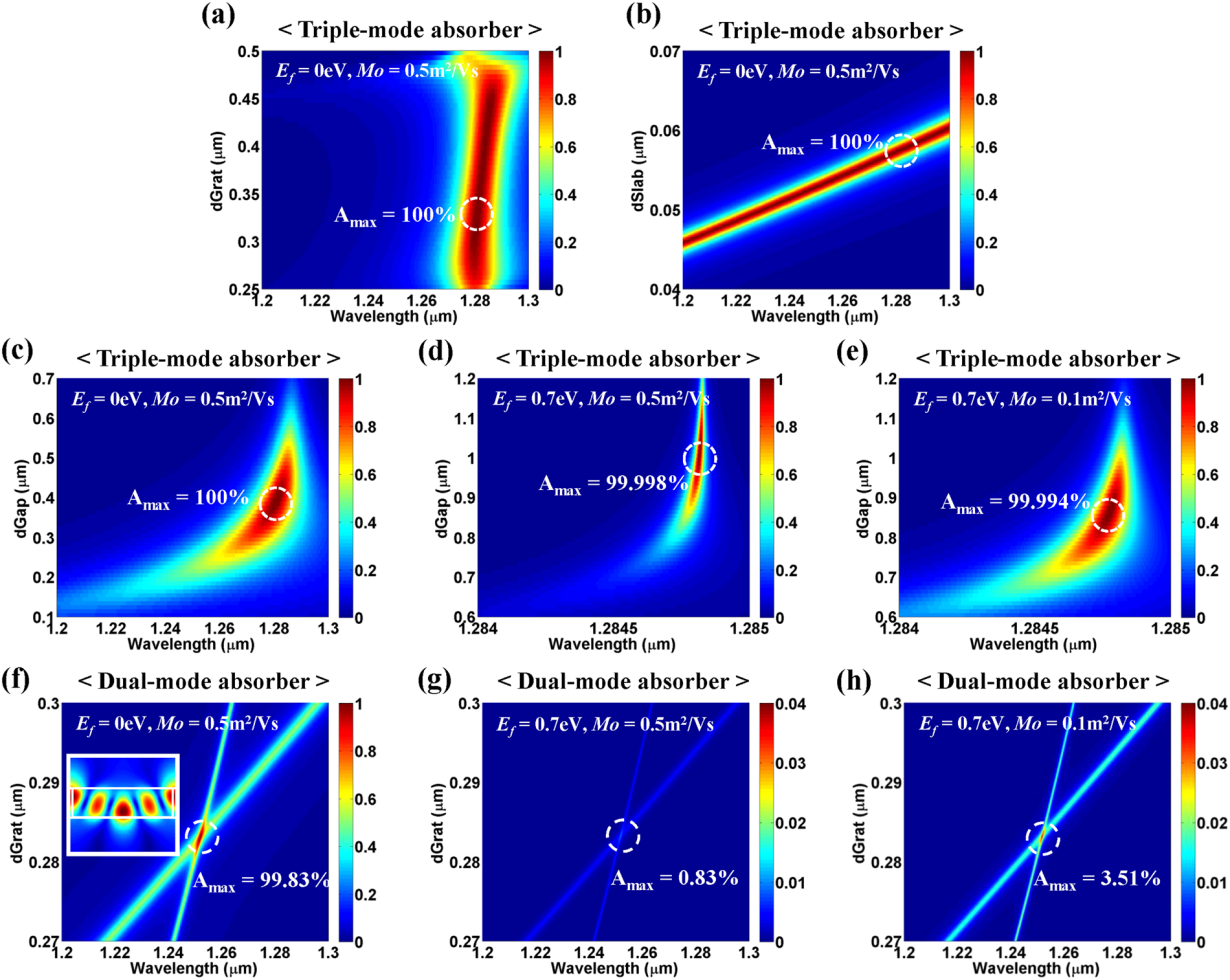
Absorption spectra as a function of (**a**) *d*_*Grat*_, (**b**) *d*_*Slab*_, and (**c**) *d*_*Gap*_ for the proposed “*triple*-*mode absorber*” when *E*_*f*_ = 0 eV, *Mo* = 0.5 m^2^/Vs. The same calculations as an (**c**) are also conducted with a different loss rate of (**d**) *E*_*f*_ = 0.7 eV, *Mo* = 0.5 m^2^/Vs, and (**e**) *E*_*f*_ = 0.7 eV, *Mo* = 0.1 m^2^/Vs. Note that the remaining parameters are the same as the optimal conditions considered in Fig. 2(f). (**f**) Absorption spectra as a function of *d*_*Grat*_ for the “*dual*-*mode absorber*” when *n*_*H*_ = 3.0, *n*_*L*_ = 1.5, *Period* = 1.0 μm, *FF* = 0.946 when *E*_*f*_ = 0 eV, and *Mo* = 0.5 m^2^/Vs (the inset indicates electric field distributions (|*E*_*y*_|) at the optimal point). The same calculations as in (**f**) are also conducted with a different loss rate of (**g**) *E*_*f*_ = 0.7 eV, *Mo* = 0.5 m^2^/Vs, and (**h**) *E*_*f*_ = 0.7 eV, *Mo* = 0.1 m^2^/Vs.

In this section, the dependency of the proposed structure on the structural parameters is investigated. Figure 5(a–c) show absorption spectra as functions of *d*_*Grat*_, *d*_*Slab*_, and *d*_*Gap*_, respectively, assuming that the remaining parameters are the same as the optimal structure considered in Fig. 2(f). Very high absorption (A > 99%) was achieved over broad ranges of the parameters (0.30 μm ≤ *d*_*Grat*_ ≤ 0.44 μm, 0.048 μm ≤ *d*_*Slab*_ ≤ 0.060 μm, and 0.37 μm ≤ *d*_*Gap*_ ≤ 0.40 μm) although the wavelength of the maximum absorption varies. This excellent tolerance to the structural parameters is partially because we have a larger number of design parameters than conditions to satisfy. More importantly, it is attributed to the strong field enhancement in the slab as mentioned earlier. Such excellent tolerance to the design parameters will be advantageous in fabrication as well as design of practical devices. Especially, the tolerance to the loss rate of the absorbing medium is quite useful for graphene-based devices because the quality of synthesized large-area graphene is hardly guaranteed and the substrate can cause unwanted doping of graphene. Even for the highly doped graphene of *E*_*f*_ = 0.7 eV with different mobilities (*Mo* = 0.5 and 0.1 m^2^/Vs), near-perfect absorption (A > 99.99%) can be obtained by adjusting *d*_*Gap*_ (Fig. 5(d–e)) although the spectral bandwidth of absorption significantly decreases owing to the reduced loss rate. For *E*_*f*_ = 0.7 eV and *Mo* = 0.5 m^2^/Vs, the loss rate corresponds to ~1/500 of the undoped graphene case of the same mobility at *λ* = 1.2805 μm (Supplementary Information). By comparing Fig. 5(d) and (e), one can see that the lower mobility case shows a wider absorption bandwidth because a lower mobility causes a higher loss in graphene (Supplementary Information). This implies that graphene of a lower mobility is more desirable for the perfect absorber application.

For comparison, the “*dual*-*mode absorber*” based on degenerate critical coupling was also designed, and its performance dependency on the structural parameters was investigated. The parameters of the designed “*dual*-*mode absorber*” with graphene of *E*_*f*_ = 0 eV and *Mo* = 0.5 m^2^/Vs are *n*_*H*_ = 3.0, *n*_*L*_ = 1.5, *Period* = 1.0 μm, *FF* = 0.946, and *d*_*Grat*_ = 0.283 μm at *λ* = 1.2518 μm as shown in Fig. 5(f), where absorption spectra are plotted as a function of *d*_*Grat*_. For the designed “*dual*-*mode absorber*”, *A*_*max*_ = 99.83% is achieved at the crossing of two resonance modes of opposite symmetries, and high absorption over 99% is obtained in the very narrow range of the grating thickness of 0.4 nm (0.2828 μm ≤ *d*_*Grat*_ ≤ 0.2832 μm), resulting in considerable difficulty in designing and fabricating practical devices. The electric field distribution at the resonance is plotted in the inset of Fig. 5(f). The “*dual*-*mode absorber*” is also very sensitive to the loss rate variation of the absorbing medium as shown in Fig. 5(g–h). The maximum achievable absorption is just *A*_*max*_ ~0.83% for *E*_*f*_ = 0.7 eV and *Mo* = 0.5 m^2^/Vs, and *A*_*max*_ ~3.51% for *E*_*f*_ = 0.7 eV and *Mo* = 0.1 m^2^/Vs.

### Wideband tunable absorption

**Figure 6:**
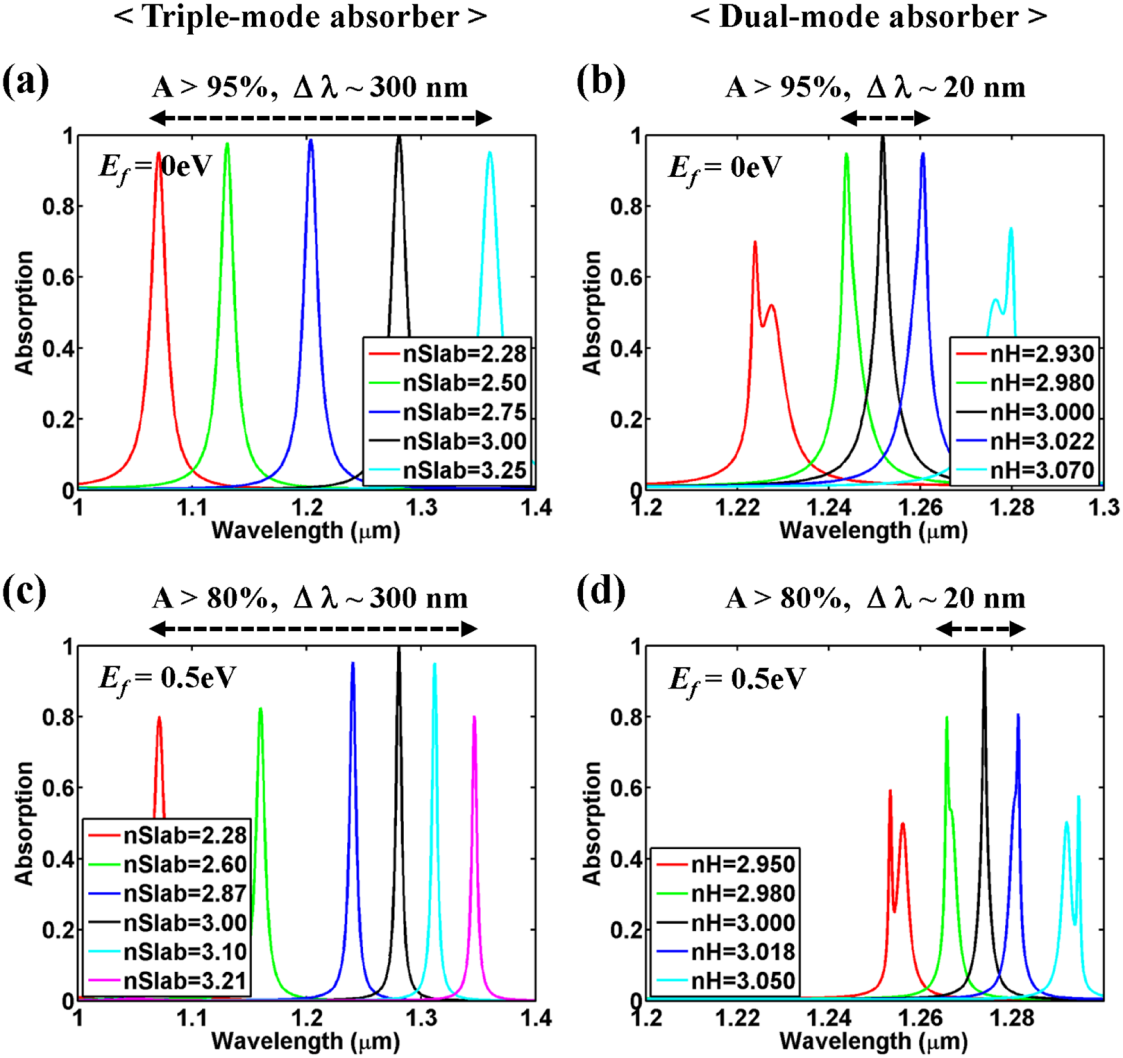
(**a**) Tunable absorption by adjusting *n*_*Slab*_ for the proposed “*triple*-*mode absorber*”, assuming that the remaining parameters are the same as the optimal conditions considered in Fig. 2(f); that is, *Period* = 0.9 μm, *FF* = 0.43, *d*_*Grat*_ = 0.32507 μm, *d*_*Gap*_ = 0.38691 μm, *d*_*Slab*_ = 0.057335 μm, and *E*_*f*_ = 0 eV. (**b**) Tunable absorption by adjusting *n*_*H*_ for the “*dual*-*mode absorber*”, assuming that the remaining parameters are the same as the optimal conditions considered in Fig. 5(f); that is, *Period* = 1.0 μm, *FF* = 0.946, *d*_*Grat*_ = 0.283 μm, and *E*_*f*_ = 0 eV. (**c**) Tunable absorption by adjusting *n*_*Slab*_ for the proposed “*triple*-*mode absorber*”, assuming that the remaining parameters are the same as the optimal conditions considered in Fig. 4(c); that is, *Period* = 0.9 μm, *FF* = 0.43, and *d*_*Grat*_ = 0.32507 μm, *d*_*Gap*_ = 0.48932 μm, *d*_*Slab*_ = 0.05649115 μm, and *E*_*f*_ = 0.5 eV. (**d**) Tunable absorption by adjusting *n*_*H*_ for the “*dual*-*mode absorber*”, assuming an optimal condition for *E*_*f*_ = 0.5 eV; that is, *Period* = 1.0 μm, *FF* = 0.968, and *d*_*Grat*_ = 0.2853 μm. In all calculations, *n*_*H*_ = 3.0, *n*_*L*_ = 1.5, and *Mo* = 0.5 m^2^/Vs.

In the proposed “*triple*-*mode absorber*”, the broadband high reflection property of the HCG is hardly affected by the design variation of the lower slab, and its absorption performance is quite insensitive to the design parameters near the optimal values as discuss earlier. Therefore, by changing *n*_*Slab*_, the absorption spectrum of the “*triple*-*mode absorber*” can be tuned over a wide wavelength range with a moderately high maximum absorption value as shown in Fig. 6(a), where tuning the peak absorption wavelength over ~300 nm is achieved by simply adjusting *n*_*Slab*_ from 2.28 to 3.25 while keeping A > 95%. For the tunable operation of the “*triple*-*mode absorber*”, electro-optic materials such as liquid crystals^22, 23^ can be used for the slab, and additional electrodes to apply a voltage will be required. In contrast, the “*dual*-*mode absorber*“ designed above shows a much narrower tuning range of ~20 nm via the variation of *n*_*H*_ from 2.980 to 3.022 for the same level of maximum absorption (A > 95%) as shown in Fig. 6(b). The wideband tunable absorption is also investigated for *E*_*f*_ = 0.5 eV, as shown in Fig. 6(c–d). Owing to the strong wavelength dependency of the loss rate (Fig. S2), the performance becomes somewhat degraded. However, the “*triple*-*mode absorber*” still has much wider and higher absorption than the “*dual*-*mode absorber*”.

## Discussion

In this work, the absorption performance of the “*triple*-*mode absorber*” composed of HCG/gap/slab/graphene layers has been investigated, in which balanced evanescent coupling between the dual nondegenerate modes of the HCG and a slab operating as a single-mode resonator is the key design issue. Although the CMT-based expressions of the perfect absorption conditions for the proposed absorber appear somewhat complex, the design process of a practical structure is remarkably simple because one can separate the optimization process of the slab waveguide and that of the HCG. In contrast, for the previously proposed “*dual*-*mode absorber*”, perfect absorption requires the conditions of *ω* = *ω*_1_ = *ω*_2_, *γ*_1_ = *γ*_1,loss_, and *γ*_2_ = *γ*_2,loss_, which implies that each of two degenerate resonance modes of opposite symmetries must have a leakage rate matched to its loss rate. Unfortunately, the degeneracy and the critical couplings of the dual modes are too restrictive to be simultaneously satisfied. Despite our best effort, a maximum absorption of *A*_*max*_ = 99.83% was obtained using the particle swarm optimization (PSO) method^24^, assuming *n*_*H*_ = 3.0, *n*_*L*_ = 1.5. In particular, it is much more difficult to satisfy the perfect absorption conditions for a practical device structure with a substrate. The substrate index *n*_*Sub*_ should be less than 0.5*n*_*H*_ to support only zeroth-order diffraction within a substrate at the degenerate resonance because any higher-order diffraction into substrate induces waste of the incident light energy. For example, the “*dual*-*mode absorber*” of *n*_*H*_ = 3.0, *n*_*L*_ = 1.5, and *n*_*Sub*_ = 1.5 can never achieve perfect absorption (Supplementary Information). On the other hand, for the proposed “*triple*-*mode absorber*” with the same substrate, perfect absorption (A > 99.99999%) can be obtained because it is less sensitive to the substrate index (Supplementary Information). For the “*triple*-*mode absorber*” with a substrate, an absorption spectrum tuning range via the index variation of the slab becomes narrower (~60 nm for A > 95%) compared to the structure without substrate (Supplementary Information).

We also investigated a more practical structure of the “*triple*-*mode absorber*” placed on a glass substrate (*n* = 1.45), where Si (*n* = 3.40) and SiO_2_ (*n* = 1.45) are used for the HCG and the gap is filled with SiO_2_ (Supplementary Information). We confirmed that perfect absorption (A > 99.99999%) can be achieved at optical wavelengths. The absorption spectrum tuning range by adjusting the index of the slab is relatively wide (~150 nm for A > 95%) despite the existence of the substrate because it still supports a considerable broadband reflection. It is obvious that the choice of the gap material does not affect the performance of the “*triple*-*mode absorber*” seriously as long as the HCG and the slab can support guided modes. We expect that the proposed “*triple*-*mode absorber*” will be applicable to photodetectors based on various absorbing media such as semiconductors as well as graphene. Furthermore, the operating wavelength of the proposed “*triple*-*mode absorber*” is scalable via a proper choice of the structural parameters.

In this work, we considered only TE wave illumination for the perfect absorber design although the basic operation principle of the “*triple*-*mode absorber*” is polarization insensitive. The polarization dependence of the present “*triple*-*mode absorber*” design mainly comes from the property of the one-dimensional (1-D) HCG. When the 1-D HCG is replaced with a two-dimensional photonic crystal slab of a square lattice, polarization-insensitive perfect absorption will be achieved for normal incidence.

## Methods

We theoretically analyzed the behavior of the proposed structure using the CMT^17–19^. We developed Matlab codes to plot the results of the CMT analysis. For numerical analysis, two-dimensional RCWA (a commercial software program, DiffractMOD) was used^20^. In the RCWA calculation, more than 300 harmonics were applied to guarantee accuracy near the resonance frequency. In all our calculations, the complex permittivity of graphene (*ε*_*g*_) was calculated using Kubo formulation based on the local random phase approximation for various *E*_*f*_^21, 22^, assuming graphene thickness of 0.34 nm, Fermi velocity of 10^6^ m/s, and mobility of 0.5 m^2^/Vs or 0.1 m^2^/Vs.

## Electronic supplementary material

Supplementary information
