# Supplementary material for: A proposal of a perfect graphene absorber with enhanced design and fabrication tolerance
Source: Sci Rep. 2017 Jul 6;7:4760. doi: 10.1038/s41598-017-04995-w (PMC5500504; doi:10.1038/s41598-017-04995-w)
Supplement: Supplementary file 1 — Supplementary information [file 41598_2017_4995_MOESM1_ESM.pdf]

< Supplementary Information >

Title: "A proposal of a perfect graphene absorber with enhanced design and fabrication tolerance"

Sangjun Lee, Thang Q. Tran, Hyungjun Heo, Myunghwan Kim & Sangin Kim*

*Corresponding Author: E-mail:sangin@ajou.ac.kr

Department of Electrical and Computer Engineering, Ajou University, Suwon, South Korea

1. Coupled Mode Theory of the proposed ‘*Triple-mode absorber*’

For the Coupled mode theory (CMT) analysis of the proposed *‘Triple-mode absorber’*, as shown in Fig. S1, we formally treated an asymmetric system of two coupled resonators, one of which is a lossless resonator with two non-degenerate resonance modes, with the leakage rates γ_1 and γ_2 and resonance frequencies ω_1 and ω_2 respectively. The second resonator supports a single lossy resonance mode with a leakage rate γ_3, a resonance frequency ω_3, and a loss rate γ_{loss}.

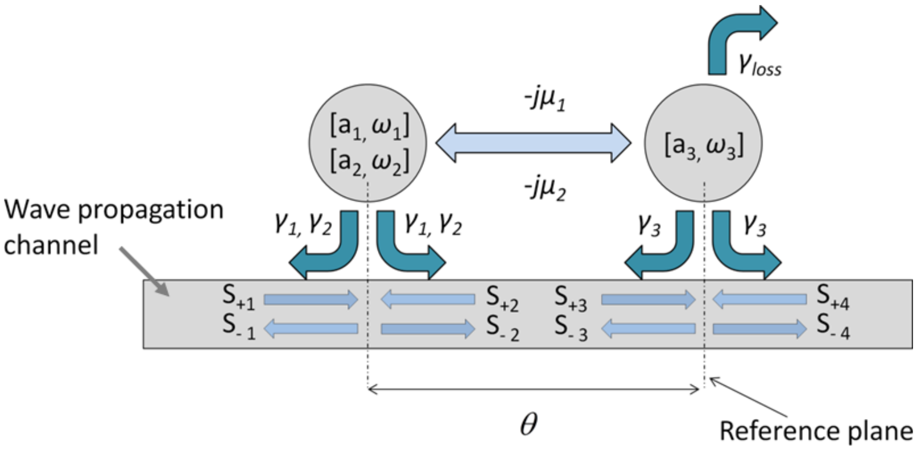

Figure S1. Theoretical model of the proposed *‘Triple-mode absorber’* for the coupled mode theory analysis, where the slab with a graphene layer is treated as a high-Q resonator (a_3) with loss, and the HCG is assumed to support two non-degenerate resonant modes (a_1 and a_2) without loss, and both direct and indirect couplings between the HCG and the slab are considered.

The dynamic of the three modes of the two resonators system could be described by the following equations [Ref. S1]:

$$\begin{cases} \dfrac{da_1}{dt} = \left(j\omega_1 - \gamma_1 \right) a_1 - j\mu_1 a_3 + d_{11}s_{+1} + d_{12}s_{+2}, \\ \dfrac{da_2}{dt} = \left(j\omega_2 - \gamma_2 \right) a_2 - j\mu_2 a_3 + d_{21}s_{+1} + d_{22}s_{+2}, \\ \dfrac{da_3}{dt} = \left(j\omega_3 - \gamma_3 - \gamma_{loss} \right) a_3 - j\mu_1 a_1 - j\mu_2 a_2 + d_3 s_{+3} + d_4 s_{+4}, \end{cases} \tag{S1}$$

with the followings describe the propagation of wave inside the propagating channel:

$$\begin{cases} s_{-1} = s_{+2} - d_{11}^* a_1 - d_{12}^* a_2, \\ s_{-2} = s_{+1} - d_{21}^* a_1 - d_{22}^* a_2, \\ s_{-3} = s_{+4} - d_3^* a_3. \\ s_{-4} = s_{+3} - d_4^* a_3, \end{cases} \tag{S2}$$

where μ_1 and μ_2 are the direct (or evanescent) coupling strengths between the modes of the first resonator and the one of the second resonator, respectively, and s_{+i} and s_{-i} are the amplitudes of the incoming and the outgoing waves respectively, with respect to the ith resonator. Assuming the propagation-induced phase difference θ between the two resonators and no returning wave after the second resonator i.e. $s_{+4} = 0$, we have:

$$\begin{cases} s_{+2} = e^{-j\theta} s_{-3} = e^{-j\theta}(s_{+4} - d_3^* a_3), \\ s_{+3} = e^{-j\theta} s_{-2} = e^{-j\theta}(s_{+1} - d_{11}^* a_1 - d_{12}^* a_2), \\ s_{+4} = 0. \end{cases} \tag{S3}$$

The energy conservation and time reversal symmetry constraints apply individually to each of the resonators as follow (assuming the scattering matrix C = $\begin{pmatrix} 0 & 1 \\ 1 & 0 \end{pmatrix}$ for both resonators): [Ref. S4]

$$\begin{cases} \begin{pmatrix} d_{11} & d_{12} \\ d_{21} & d_{22} \end{pmatrix}^T \begin{pmatrix} d_{11} & d_{12} \\ d_{21} & d_{22} \end{pmatrix} = 2\begin{pmatrix} \gamma_1 & \gamma_0 \\ \gamma_0^* & \gamma_2 \end{pmatrix} \\ \begin{pmatrix} 0 & 1 \\ 1 & 0 \end{pmatrix}\begin{pmatrix} d_{11}^* & d_{12}^* \\ d_{21}^* & d_{22}^* \end{pmatrix} + \begin{pmatrix} d_{11} & d_{12} \\ d_{21} & d_{22} \end{pmatrix} = 0 \end{cases} \tag{S4}$$

and

$$
\begin{cases}
\begin{pmatrix} d_3 \\ d_4 \end{pmatrix}^T \begin{pmatrix} d_3 \\ d_4 \end{pmatrix} = 2\gamma_3 \\
\begin{pmatrix} 0 & 1 \\ 1 & 0 \end{pmatrix} \begin{pmatrix} d_3^* \\ d_4^* \end{pmatrix} + \begin{pmatrix} d_3 \\ d_4 \end{pmatrix} = 0
\end{cases}
\tag{S5}
$$

Assuming mirror symmetry for both resonators, and also assuming that the two resonance modes of the first resonator has opposite symmetry, where the first mode and second mode are of even and odd symmetry respectively, we obtain:

$$
\begin{cases}
d_{ij} = \sqrt{\gamma_i} e^{j\theta_{ij}} \\
\gamma_0 = 0 \\
\theta_{21} = \theta_{11} \\
\theta_{22} = \theta_{12} + \pi
\end{cases}
\tag{S6}
$$

for the first resonator, and

$$
\begin{cases}
d_3 = d_4 = e^{j\varphi} \sqrt{2 / \tau_3}, \\
\varphi = \theta_{11},
\end{cases}
\tag{S7}
$$

for the second resonator. For simplicity, we assumed $\varphi = \theta_{11}$, since any residual phase differences would be included in the propagating-induced phase difference θ.

Combining equations (S1) through (S7) and solve the obtained linear system, the complex transmission and reflection coefficients of the system are as follow:

$$
\begin{cases}
r = \dfrac{s_{-1}}{s_{+1}} = \dfrac{N1}{D}, \\
t = \dfrac{s_{-4}}{s_{+1}} = \dfrac{N2}{D},
\end{cases}
$$

where

$$
\begin{aligned}
N1 = {} & \mathrm{e}^{2j\theta} \left[\mu_1^2 \gamma_2 - \mu_2^2 \gamma_1 - 2j\mu_1\mu_2 \sqrt{\gamma_1}\sqrt{\gamma_2} + \left(\gamma_2(-\omega + \omega_1) + \gamma_1(\omega - \omega_2) \right) \left(-j\gamma_{loss} - j\gamma_3 + \omega - \omega_3 \right) \right] \\
& -2\mathrm{e}^{j\theta} \sqrt{\gamma_3} \left(\mu_2 \sqrt{\gamma_2} (\gamma_1 - j(\omega - \omega_1)) + \mu_1 \sqrt{\gamma_1} (j\gamma_2 + \omega - \omega_2) \right) + \gamma_3 (j\gamma_1 + \omega - \omega_1)(j\gamma_2 + \omega - \omega_2), \\
N2 = {} & \mathrm{e}^{2j\theta} \sqrt{\gamma_3} \left(\mu_2 \sqrt{\gamma_2} (\gamma_1 + j(\omega - \omega_1)) + \mu_1 \sqrt{\gamma_1} (j\gamma_2 - \omega + \omega_2) \right) + \sqrt{\gamma_3} \left(-\mu_2 \sqrt{\gamma_2} (\gamma_1 - j(\omega - \omega_1)) + \mu_1 \sqrt{\gamma_1} (j\gamma_2 + \omega - \omega_2) \right) \\
& +\mathrm{e}^{j\theta} \left[-\gamma_{loss} (\gamma_1\gamma_2 + (\omega - \omega_1)(\omega - \omega_2)) + j \left(\mu_2^2 (\omega - \omega_1) + \mu_1^2 (\omega - \omega_2) - \left(\gamma_1\gamma_2 + (\omega - \omega_1)(\omega - \omega_2) \right)(\omega - \omega_3) \right) \right], \\
D = {} & \mathrm{e}^{2j\theta} \left[\mu_2^2 \left(\gamma_1 + j(\omega - \omega_1) \right) + \mu_1^2 \left(\gamma_2 + j(\omega - \omega_2) \right) + \left(\gamma_2 + j(\omega - \omega_2) \right) \left(\gamma_1 + j(\omega - \omega_1) \right) \left(\gamma_{loss} + \gamma_3 + j(\omega - \omega_3) \right) \right] \\
& +2\mathrm{e}^{j\theta} \sqrt{\gamma_3} (\mu_2 \sqrt{\gamma_2} (\gamma_1 + j(\omega - \omega_1)) + \mu_1 \sqrt{\gamma_1} (-j\gamma_2 + \omega - \omega_2)) + j\gamma_3 (\gamma_2 (\omega - \omega_1) - \gamma_1 (\omega - \omega_2)).
\end{aligned}
$$

Assuming that $\omega_1 = \omega_{\text{even}} < \omega < \omega_{\text{odd}} = \omega_2$, or $\omega - \omega_1 > 0 > \omega - \omega_2$, and solving for perfect absorption conditions, we have, at the resonance frequency $\omega = \omega_0$, for t=0:

$$\begin{cases} (\omega_0 - \omega_1)(\omega_0 - \omega_2) + \gamma_1\gamma_2 = 0, \\ \left(\mu_1(\omega_0 - \omega_2) + \mu_2\sqrt{\gamma_1\gamma_2}\right) + 2\sqrt{\gamma_1\gamma_3}\left(\gamma_2\cos\theta - (\omega_0 - \omega_2)\sin\theta\right) = 0, \end{cases} \tag{S8}$$

and for r=0:

$$\begin{cases} \left[(\omega_0 - \omega_3)\gamma_1\left((\omega_0 - \omega_2)^2 + \gamma_2{}^2\right) + (\omega_0 - \omega_2)\left(-\mu_2{}^2\gamma_1 + \mu_1{}^2\gamma_2 - 2\gamma_1\gamma_2\gamma_3\right)\right]\cos\theta \\ +\left[2(\omega_0 - \omega_2)\mu_1\mu_2\sqrt{\gamma_1\gamma_2} + \gamma_{loss}\gamma_1\gamma_2{}^2 + (\omega_0 - \omega_2)^2\gamma_1(\gamma_{loss} + 2\gamma_3)\right]\sin\theta \\ -2\sqrt{\gamma_1\gamma_3}(\omega_0 - \omega_2)\left(\mu_1(\omega_0 - \omega_2) + \mu_2\sqrt{\gamma_1\gamma_2}\right) = 0, \\ \left[\gamma_{loss}\gamma_1\left((\omega_0 - \omega_2)^2 + \gamma_2{}^2\right) + (\omega_0 - \omega_2)\mu_1\mu_2\sqrt{\gamma_1\gamma_2} + \gamma_1\gamma_2{}^2\gamma_3\right]\cos\theta \\ -\left[(\omega_0 - \omega_3)\gamma_1\left((\omega - \omega_2)^2 + \gamma_2{}^2\right) + (\omega_0 - \omega_2)\left(-\mu_2{}^2\gamma_1 + \mu_1{}^2\gamma_2 + 2\gamma_1\gamma_2\gamma_3\right)\right]\sin\theta \\ +2\gamma_2\sqrt{\gamma_1\gamma_3}\left(\mu_1(\omega_0 - \omega_2) + \mu_2\sqrt{\gamma_1\gamma_2}\right) = 0. \end{cases} \tag{S9}$$

Assuming ω_3, γ_3, γ_{loss} are optimizable variables and the remaining symbols are predetermined design parameters, we obtained the following perfect absorption conditions:

$$(\omega_0 - \omega_1)(\omega_0 - \omega_2) + \gamma_1\gamma_2 = 0, \tag{S10}$$

$$\theta = (2n+1)\pi, \tag{S11}$$

$$\sqrt{\gamma_3} = \frac{\mu_1(\omega_0 - \omega_2) + \mu_2\sqrt{\gamma_1\gamma_2}}{2\gamma_2\sqrt{\gamma_1}}, \tag{S12}$$

$$\gamma_{loss} = \frac{-2\mu_1\mu_2\sqrt{\gamma_1\gamma_2}(\omega_0 - \omega_2) + 2\gamma_1\gamma_2{}^2\gamma_3}{\gamma_1\left((\omega_0 - \omega_2)^2 + \gamma_2{}^2\right)}, \tag{S13}$$

and

$$\omega_3 = \omega_0 - \frac{(\omega_0 - \omega_2)\left(\mu_2{}^2\gamma_1 - \mu_1{}^2\gamma_2 - 2\gamma_1\gamma_2\gamma_3\right)}{\gamma_1\left((\omega_0 - \omega_2)^2 + \gamma_2{}^2\right)}. \tag{S14}$$

Since $\sqrt{\gamma_3}$ is real and positive, the following condition has to be satisfied:

$$\omega_0 - \omega_2 > -\frac{\mu_2\sqrt{\gamma_1\gamma_2}}{\mu_1}. \tag{S15}$$

Also, for the solutions to equation (S10) to be real, the following condition has to be satisfied:

$$(\omega_1 - \omega_2)^2 > 4\gamma_1\gamma_2 \tag{S16}$$

We may have two, one, or no solutions satisfying the conditions (S15) and (S16) depending on the ratio of μ_1/μ_2.

2. Optical constants of graphene

Optical properties of graphene can be described with a complex permittivity, and its imaginary part is directly related to the loss rate in graphene. In Fig. S2, a real part of a conductivity (σ_G) and an imaginary part of the permittivity (ε_G) of graphene are plotted, which are calculated from Kubo formula for vaious Fermi-levels (E_f = 0, 0.3, 0.5, 0.7eV) and mobilities (Mo = 0.5, 0.1m^2/Vs). A higher E_f induces a decrease in loss rate. For E_f = 0.5eV and 0.7eV, for instances, the loss rates decrease to ~1/3 and ~1/500 of the undoped graphene's value, respectively, at λ = 1.2805μm and Mo = 0.5m^2/Vs. As for the mobility dependency of the loss, over all, the higher mobility brings out the lower loss, which becomes noticeable when E_f is sufficiently high so that the graphene becomes metal-like. For example, for λ = 1.2 ~ 1.3 μm, the mobility dependency is very strong for E_f = 0.7eV, while it is negligibly weak for E_f = 0.5eV.

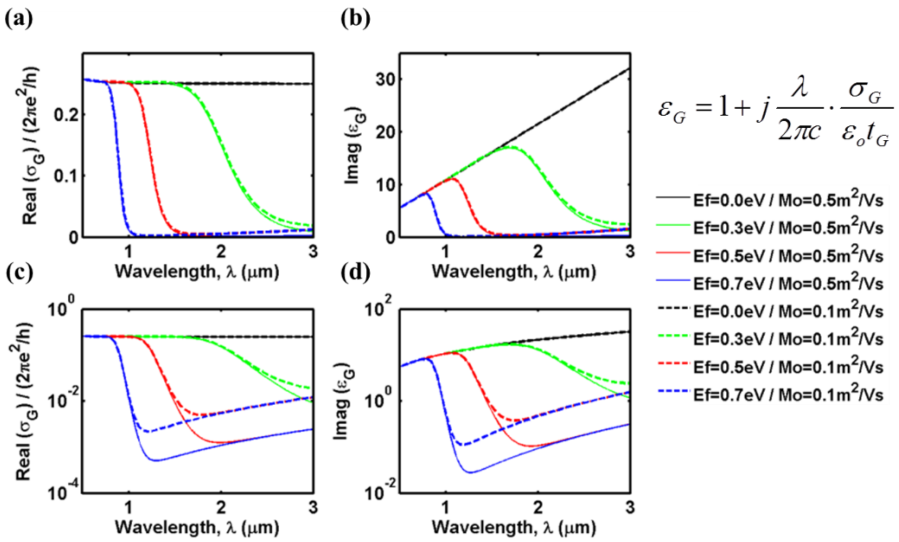

Figure S2. (a) Real part of conductivity and (b) Imaginary part of permittivity of graphene for various Fermi-levels and mobilities (on a linear scale). (c) and (d) are the same plots as (a) and (b) on a log scale, respectively.

3. Perfect absorber with substrate of $n_{Sub} = 0.5n_H$

As previously noted, perfect absorption conditions for the previously proposed *'Dual-mode absorber'* are excessively restrictive to be easily satisfied. In particular, it is much more difficult to satisfy the conditions for a practical device structure with a substrate. The substrate index n_{Sub} should be smaller than $0.5n_H$ to support only zeroth-order diffraction within a substrate at the degenerative resonance because any higher-order diffraction into the substrate induces the waste of the incident light energy. For example, the *'Dual-mode absorber'* with $n_H = 3.0$, $n_L = 1.5$, $n_{Sub} = 1.5$ can never achieve perfect absorption (Fig. S3(a)).

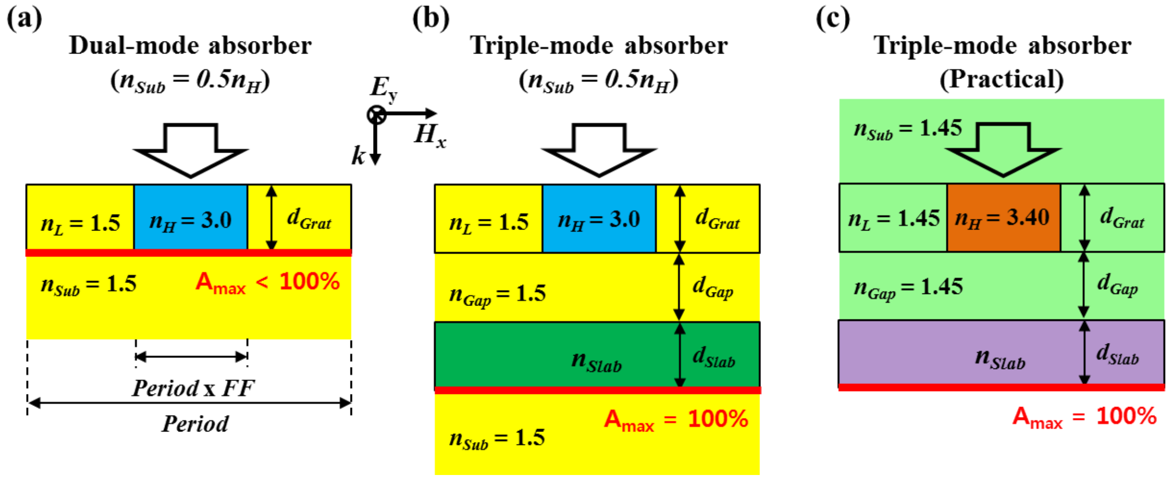

Figure S3. Schematic of (a) *'Dual-mode absorber'* and (b) *'Triple-mode absorber'* with substrate of $n_{Sub} = 0.5n_H$. (c) Schematic of practical *'Triple-mode absorber'* composed of Si and SiO_2. The red solid lines indicate monolayer graphene of 0.34m thickness as an absorbing medium. In all the RCWA calculations, $E_f = 0$ eV, $Mo = 0.5$ m^2/Vs are assumed.

On the other hand, for our proposed *'Triple-mode absorber'* with the same substrate (Fig. S3(b)), perfect absorption can occur because it is less sensitive to substrate index, as shown in Fig. S4. We confirmed the absorber's perfect absorption (A > 99.99999%) under the optimal condition $n_{Slab} = 3.0$, $\lambda = 1.5161$ µm (marked by dashed circle). Similar to the absorber without substrate (Fig. 2(f)), as expected, the wavelength of perfect absorption exactly matches to the main transmission dip of the HCG alone case (Fig. S4(c)). Owing to the effect the substrate, an absorption spectrum tuning range via the index variation of the slab gets narrower (~60nm for A > 95%, as seen in Fig. S4(d)), compared to the structure without substrate.

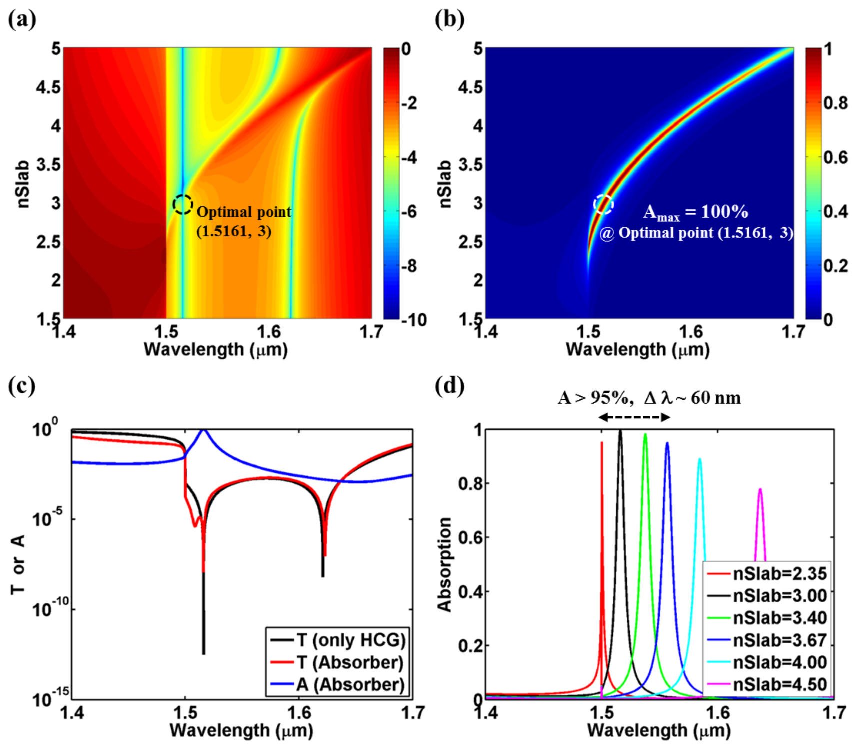

Figure S4. (a) Transmission spectra and (b) Absorption spectra as a function of n_{Slab} for the proposed *'Triple-mode absorber'* with substrate of n_{Sub} = $0.5n_H$. (c) Transmission or absorption spectra for the optimized absorber (n_{Slab} = 3) and the lossless HCG alone. (d) Tunable absorption by adjusting n_{Slab}. In all the calculations, n_H = 3.0, n_L = n_{Gap} = n_{Sub} = 1.5, *Period* = 1 μm, *FF* = 0.19, d_{Grat} = 0.509745 μm, d_{Slab} = 0.0195292 μm, and d_{Gap} = 0.85296 μm.

3. Practical perfect absorber

Figure S3(c) shows the practical *'Triple-mode absorber'* placed on glass substrate (*n* = 1.45), where Si (*n* = 3.40) and SiO_2 (*n* = 1.45) are used for the HCG, and the gap is filled with SiO_2. Since the HCG is buried in a semi-infinite SiO_2, this structure can be fabricated more easily: the upper part (the HCG and the slab) is fabricated first on a quartz (or glass) and a separately prepared (synthesized or exfoliated) graphene layer can be just transferred to the top of the slab. We investigated transmission and absorption spectra as a function of slab index n_{Slab} for the practical structure, as shown in Fig. S5(a) and S5(b) respectively. Perfect absorption (A > 99.99999%) are achieved under the optimal condition n_{Slab} = 3.4, λ = 1.5637 μm (marked by dashed circle). As seen in Fig. S5(d), the absorption spectrum tuning range by adjusting of slab index is relatively wide (~150 nm for A > 95%), despite the existence of a substrate because it still supports a considerable broadband reflection.

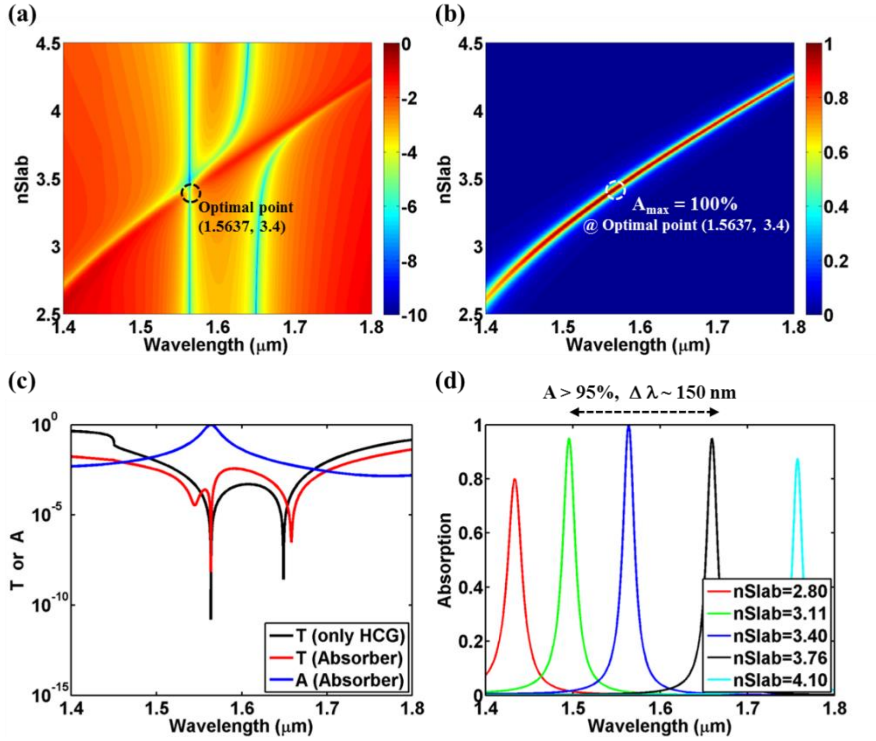

Figure S5. (a) Transmission spectra and (b) Absorption spectra as a function of n_{Slab} for the practical *'Triple-mode absorber'* composed of Si and SiO_2. (c) Transmission and absorption spectra for the optimized absorber (n_{Slab} = 3.4) and the lossless HCG alone. (d) Tunable absorption by adjusting of n_{Slab}. In all the calculations, n_H = 3.4, $n_L = n_{Gap} = n_{Sub}$ = 1.45, *Period* = 1 μm, *FF* = 0.46, d_{Grat} = 0.307244 μm, d_{Slab} = 0.047891 μm, and d_{Gap} = 0.736338 μm.

References for Supplementary materials:

[Ref. S1] Haus, H. A. *et al.* Coupled Mode Theory *Proceedings of the IEEE,* **19**(10) (1991).

[Ref. S2] Piper, J. R. *et al.* Total absorption by degenerate critical coupling. *Appl. Phys. Lett.* **104**, 251110 (2014).

[Ref. S3] Suh, W. *et al.* All-pass transmission or flattop reflection filters using a single photonic crystal slab. *Appl. Phys. Lett.* **84**, 4905 (2004).

[Ref. S4] Suh, W. *et al.* Temporal coupled-mode theory and the presence of non-orthogonal modes in lossless multimode cavities. *IEEE J. of Quantum Electron.* **40**(10) (2004).
